# Increased ROM and high patient satisfaction after open arthrolysis: a follow-up-study of 43 patients with posttraumatic stiff elbows

**DOI:** 10.1186/s12891-016-0928-8

**Published:** 2016-02-12

**Authors:** Petter Morten Pettersen, Joakim Eriksson, Hallgeir Bratberg, Lars Eldar Myrseth, Lise Grete Bjørnstad, Marte Johansen, Torstein Husby

**Affiliations:** Mail Box 1078, Blindern, 0316 Oslo Norway; Orthopedic Department, Section for Upper extremity surgery, Oslo University Hospital, Oslo Universitetssykehus HF, Mail Box 4950, Nydalen, 0424 Oslo Norway; Physiotherapy Department, Oslo Universitetssykehus HF, Mail Box 4950, Nydalen, 0424 Oslo Norway; Radiological Department, Oslo Universitetssykehus HF, Mail Box 4950, Nydalen, 0424 Oslo Norway

**Keywords:** Stiff elbow, Arthrolysis, Contracture, Capsulectomy

## Abstract

**Background:**

Posttraumatic stiffness of the elbow is a common finding after elbow trauma. Restoration of motion in the posttraumatic stiff elbow is difficult, time consuming, and requires high patient compliance. We have evaluated the long-term effect of an open elbow arthrolysis in the posttraumatic stiff elbow.

**Methods:**

We evaluated 43 patients (14 women, 29 men) with a median age of 47(16–78) years operated with open arthrolysis for a posttraumatic stiff elbow. The median follow-up time was 41(12–204) months. The patients were hospitalized median 12(4–14) days, with daily physiotherapy and NSAID. 36 patients tolerated continuous passive motion (CPM) for 11(0–42) days. 35 patients had a well-functioning brachial plexus anesthesia for median 7(1–18) days. We used the paired 2-tailed T-test in our statistical analysis.

**Results:**

Preoperatively the patients had a median flexion of 110(30–160)°, extension 40(10–90)°, and the total flexion-extension sector (F/E) was 50(0–110)°. At follow-up the patients had a median flexion of 132(75–151)° and extension of 23(8–84)°, which indicate a median gain of 42(−50–114)°. The subjective functional scores (Mayo Elbow Score, EQ5D, Q-Dash, and VAS for pain) were satisfying, and most of the patients (81 %) would have done the operation once again knowing the outcome. We had 5 temporary ulnar neuropraxias, one became permanent and in addition ankylotic, one temporary radial neuropraxia, two superficial wound infections, and one transient hematoma.

**Conclusion:**

Open arthrolysis of the posttraumatic stiff elbow is associated with reliable clinical and functional long-term outcomes.

## Background

An elbow contracture is disabling and common posttraumatic sequelae [1, 2]. An otherwise normal hand function will be grossly limited combined with a stiff elbow [1]. Elbow flexion is more important than extension and the goal for treatment is to restore a functional range of motion (ROM) between 30-130°. Modern equipment such as mouse and keyboard may require more pronation, while holding a cell phone may require a flexion of more than 120 ° [3]. The cause of stiffness is both extra- and intraarticular scarring and heterotopic bone formation [4]. Arthrolysis and capsulectomy of the elbow may be done arthroscopically or in an open fashion [5, 6]. The proximity to vulnerable nerves and vessels may make an endoscopic procedure challenging, due to scarring and secondary changes after earlier injuries and operations [6]. Indications for an open release may still be: elbows requiring an ulnar nerve release, hardware removal, proximal radio-ulnar joint surgery, gross osteoarthritis, and necessarily surgeons with limited arthroscopic experience. For this reason open arthrolysis still has an indication. After arthrolysis of the elbow there is a strong tendency towards restiffening of the joint [2]. The purpose of our study was to present the joint function and patient satisfaction in a long-term study on open elbow arthrolysis. Our hypothesis was that the open arthrolysis of the stiff elbow is associated with good long-term results and that severe contractures should not necessarily represent an absolute contraindication for surgery.

**Fig. 1:**
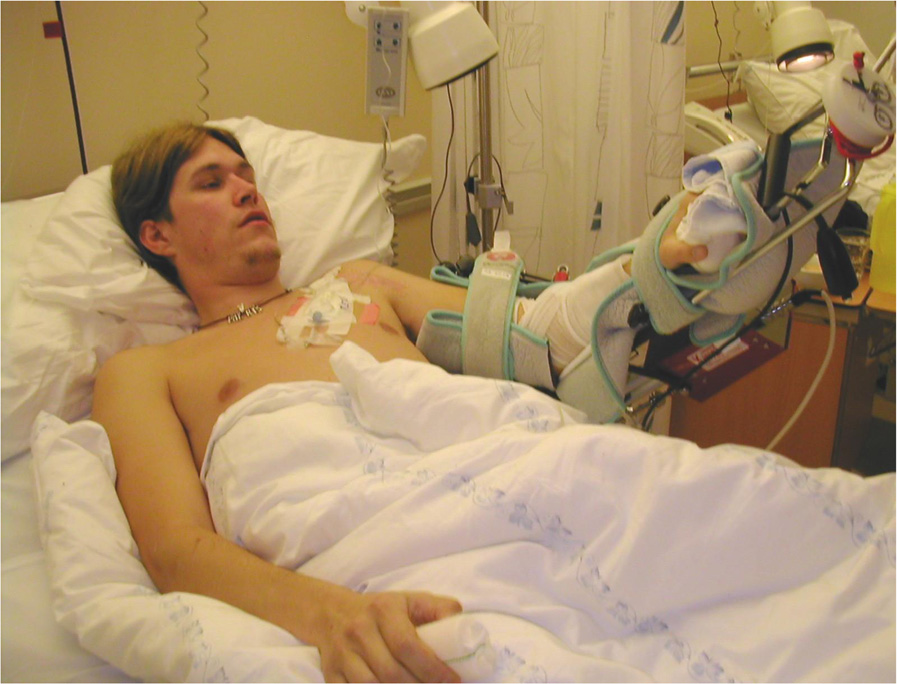
A young man operated with open elbow arthrolysis treated with postoperative continuous passive motion and plexus anesthesia. (The patient has provided consent for the publication of this image)

## Methods

All patients operated with an open elbow arthrolysis for a posttraumatic stiff elbow at our department between 1995 and 2011 were invited to participate in the study. A written informed consent for participation in the study was obtained from all participants. Of a total of 56 patients, 43 patients were able and motivated for participation in the study, 29 men and 14 women. The median time between the initial incidence and the open arthrolysis was 21(2–264) months. 24 patients were operated on the dominant side. All patients were examined at follow-up with x-rays and clinical evaluation by a senior radiologist, a physiotherapist and an orthopedic surgeon (one of the authors). We used the Kellgren & Lawrence classification for assessment of osteoarthritis [7]. Descriptive data and demographics are documented in Tables 1 and 2. Seven patients had already had complications due to the initial injury (6 nerve injuries, 2 infections, 1 CRPS). Prior to our intervention 12 patients had underwent an earlier unsuccessful arthrolysis at a local hospital. In fact, this was the reason for referral to our hospital. 8 patients in our material reported preoperative locking. 22 patients had osteosynthesis hardware left in the elbow prior to our arthrolysis. The ultimate preoperative goal for the release was a normal flexion/extension, but this was adjusted individually according to the severity of the contracture. When the patient had severe rotational contractures we aimed for a release of the proximal radioulnar joint and its periarticular space. 37 patients were operated under general anesthesia, 7 had a brachial plexus anesthesia. Regardless of earlier scars we commenced the procedures with a lateral or extended lateral approach. When achieving a satisfactory arthrolysis/capsulectomy both anterior and posterior through this access and the intraoperative ROM was good, we finalized the operation at this point (Table 3). In two patients we found it mandatory with an additional medial approach, securing the ulnar nerve and making a medial arthrolysis/capsulectomy as well. We did not routinely make a decompression of the ulnar nerve, and did not excise the radial head in any patients. Our most common procedure was a posterior and anterior capsulectomy combined with soft tissue release and resection of new bone formation.

**Table 1:** Demographic data of 43 patients operated with open arthrolysis

Variable	Median (Range)
Age (years)	47 (16–78)
Duration of symptoms prior to surgery (months)	21 (2–264)
Follow-up time (months)	41 (12–204)
Preoperative sick leave (months)	1 (0–54)
Preoperative pain (VAS, 0–100)^a^	40 (0–100)

^a^The patients were asked to estimate their preoperative VAS score retrospectively

**Table 2:** Initial injury of 43 patients operated with an open arthrolysis

Type of injury	Number of patients
Primary dislocati	9
Initial fractures	35
- Radial head	10
- Intraarticular distal humerus	6
- transverse of the humerus	4
- olecranon	3
- combinations	12
- open fractures	4

**Table 3:** Procedures performed in arthrolysis on 43 patients

Surgical techniques	Number of patients
Posterior and anterior capsulectomy + removal of bone spurs	17
Posterior and anterior capsulectomy	12
Anterior capsulectomy	8
Posterior capsulectomy	3
Anterior capsulectomy + removal of bone spurs	2
Posterior capsulectomy + removal of bone spurs	1

The patients were hospitalized for median 12(4–14) days. All the patients had daily physiotherapy during the stay. The patients were given 25 mg Indomethacin orally 3 times daily for 10 days. Continuous postoperative brachial plexus anesthesia was successful in 35 cases with a mean endurance of 9(SD 1) days (Fig. 1). 8 patients had an insufficient effect of the brachial anesthesia. 36 patients had Continuous Passive Motion (CPM, Kinetec) postoperatively with a median duration of 12(4–42) days. Most patients tolerated the CPM machine during the night as well. As much as the plexus anesthesia permitted, the patients were taught daily active exercises in cooperation with the physiotherapist. A few patients used the Kinetec machine as outpatients after leaving the hospital.

For our statistical analysis of the pre- and postoperative mobility and subjective scores we used the paired T-test.

### Ethics

The regional Committee for Medical Ethics approved the study, and all patients signed a written informed consent before inclusion. The members of the Ethical Committee were as follows: Stein Opjordsmoen Ilner (Chairman), Grete Dyb (co-chairman), Ingun Sletnes, Anne-Mari Torgersen, Berit Herlofsen, Kjetil Fretheim, Frank Oterholt, Ellen Beccer Brandvold, Gerd-Berit Odberg, all affiliated to the Regional committee for Medical Ethics in Science, Sør-Øst B, Mailbox 1130 Blindern, 0318 Oslo, Norway (http://helseforskning.etikkom.no)

## Results

### Mobility

At follow-up the patients experienced a significant increase in flexion and extension (F/E) arc and an improvement in rotation as well, compared to the preoperative status. The median gain in F/E arc was 42 (−50–114)° and the total rotational gain was 9 (−140–107)° (Table 4). 11 patients had a substantial improvement (>50°) in rotation at follow-up. All these patients had a focal bony blocking due to secondary ossifications as an explanation for the reduced rotation preoperatively.

**Table 4:** Key results in arthrolysis in 43 patients. Figures are in median (range). The *P*-values refer to the preoperative compared to follow-up measures (The paired 2-tailed T-test)

Variable	Preoperative	Peroperative	Follow-up	*P*-value
Flexion	110 (30–160)°	130 (110–160)°	132 (75–151)°	<0.01
Extension	40 (10–90)°	15 (5–70)°	23 (8–84)°	<0.01
F/E arc	50 (0–110)°	120 (60–160)°	106 (0–144)°	<0.01
Pronation	70 (0–90)°	70 (0–90)°	72 (5–86)°	0.03
Supination	60 (0–96)°	78 (0–96)°	69 (−26-96)°	0.24
Total rotation	120 (0–182)°	140 (0–182)°	139 (0–174)°	0.03

### Restiffening

The tendency towards restiffening of the elbow postoperatively was demonstrated by the fact that we had 17(SD 4)° reduction in the F/E arc during the follow-up period compared to the peroperative findings (Table 4).

### Subjective scores

Functional elbow scores at follow-up are documented in Table 5. Subjectively 4 patients considered the arthrolysis as excellent, 18 as good, 14 as satisfactory, and 7 as bad. The patients were asked to recall their estimated preoperative pain score at follow-up (Table 5). At follow-up patients with a F/E arc >100° (*n* = 26) had an EQ5D score of 0.76(SD 0.03), while patients with F/E arc <100° (*n* = 17) scored 0.62(SD 0.05). The respective figures for EQ5D-VAS were 79(SD 3), and 68(SD 4) (*p* < 0.05). For the Q-DASH score the same trend was notified (*p* = 0.01). As expected the functional scores improved with increased ROM.

**Table 5:** Subjective functional scores at follow-up. Figures are in median (range)

Variable	At follow-up
Mayo Elbow score (100=excellent)	85 (50–100
EQ5D score (Max = 1.00)	0.76 (0.05–1.0)
EQ5D-VAS	76 (36–100)
Quick-DASH score	20 (2–68)
Pain (VAS, 100 = worst pain)	
- At rest	11 (0–60)
- At light activity	15 (0–90)
- At heavy activity	40 (0–100)

35 patients would have done the operation once again, knowing the outcome.

### Osteoarthritis

The 19 patients with little or no osteoarthritis increased their mean F/E arc with 51(SD 6)°. 17 patients with moderate osteoarthritis (grade I and II) increased their F/E arc with 43(SD 6)°, and 6 patients with grade III osteoarthritis increased the F/E arc with 30°. One patient with grade IV osteoarthritis decreased the F/E arc with 50° and resulted in a total failure (ankylosis). There was a general increased degree of osteoarthritis from the preoperative findings to the follow-up x-rays. The numbers of patients with no osteoarthritis decreased from 19 to 10, the degree of minimal osteoarthritis increased from 17 to 23, moderate osteoarthritis from 6 to 8, and severe osteoarthritis from 1 to 2 patients, respectively. At follow up 32 patients had bony spurs or heterotopic bone formation, and 2 patients had calcific deposits in the capsule.

### Complications

The total complication rate was 47 %: 5 ulnar neuropraxias resolved (1 after antepositioning), 1 ulnar neuropraxias became permanent and the same elbow became ankylotic as well. We had 1 temporary radial neuropraxia (resolved), 2 transient superficial wound infections, 1 transient hematoma, and 3 minor temporary scar problems. All patients with neuropraxias of the ulnar nerve had a preoperative flexion <110°. 8 patients had a secondary mobilization of the elbow under general anesthesia in the early postoperative period due to rapid restiffening of the elbow. 4 patients had a new fall on the elbow (contusion without fractures or dislocations) during the follow-up period.

At follow-up all elbows were stable. The median postoperative sick leave was 1(0–54) months, varying substantially according to workload.

## Discussion

A systematic review of surgical treatment of posttraumatic elbow stiffness includes 21 articles with open surgical procedures [8]. When comparing the results from our study with this meta-analysis we find that the mean gain in F/E arc was lower in our study (43 vs. 51°). This may be explained by a higher mean age in our population (48 vs. 38 years) and a longer follow-up time. Patient selection, severity of the contractures and osteoarthritis may explain the differences as well. Some of our patients did not comply very well and this may also have influenced the end results. Considering functional scores and complication rates our findings are in accordance with others [2, 8–15].

The question of active versus passive mobilization in the early postoperative period must be addressed. With continuous brachial plexus anesthesia the patients tolerate passive mobilization in the CPM machine, even when asleep. Postoperative restiffening of the elbow is a major problem after arthrolysis of the elbow [16]. In accordance with others [2] we found that the patients are likely to lose mobility compared to the peroperative ROM. Higgs et al. use the CPM machine for 48 h postoperatively with comparable results as ours [2]. We experience that the pain postoperatively is substantial for about one week. Due to this, we find it convenient for the patient’s comfort to prolong both the plexus anesthesia and CPM treatment for 10–12 days postoperatively. We stopped the CPM treatment when the physiotherapist considered the patient to be in a steady state, and the patients were then dismissed. We did not record the possible changes in ROM immediately after the demounting of the CPM machine. In selected cases an outpatient CPM machine will be required for a period in addition to this.

In the case of rapid restiffening, some authors advocate early postoperative gentle mobilization [2, 4, 12, 17, 18]. We do not think this is obligatory, but it might be an option in selected cases with recurrent contractures in the early postoperative period.

Although not significant, our findings indicate that a lack of osteoarthritis positively correlates to a durable increased ROM in the elbow after arthrolysis. On the contrary, gross elbow osteoarthritis preoperatively reduces the long-term outcome from an arthrolysis. This is in accordance with Urbaniak et al. [19]. Honest information concerning realistic expectations should be given preoperatively to all the patients with stiff elbows.

Severe elbow contractures due to significant osteoarthritis seem not to be an absolute contraindication for surgery, as long as there is some cartilage resources left in the joint space. In cases with advanced osteoarthritis in the elbow an open arthrolysis may not be indicated. In such cases a total elbow arthroplasty might be considered.

We recorded 1 patient with two serious complications, a permanent lesion to the ulnar nerve in addition to ankylosis of the elbow. This was a reoperation of an almost ankylotic elbow, which should have been treated differently with a medial opening securing the ulnar nerve peroperatively. This emphasizes the importance of proper patient selection [20].

Our complication rate was high, but most of the complications were minor and transient and did not influence the long-term outcome. These findings are in accordance with other studies of open arthrolysis of the elbow [8, 21].

The question of open versus arthroscopic arthrolysis is important. Comparing complication rates between the two methods are difficult due to the lack of comparative studies. There are studies with low complication rates with both techniques [5, 10, 19, 22, 23], but the selection of patients varies substantially in these materials. Arthroscopic capsular release is a challenging technique due to the anatomical proximity of neurovascular structures in the elbow. In addition the joint space may be limited due to scarring and secondary osteoarthritis. Dealing with the ulnar nerve and heterotopic bone formation might be challenging with the arthroscopic technique [6, 24–26]. Some papers report inferior results concerning ROM after arthroscopic compared to the open elbow arthrolysis [6, 27]. This may indicate that the endoscopic technique is technically demanding and has a slow learning curve compared to the relatively straightforward open procedure. Considering the potentially increased complication risk with the arthroscopic technique [28, 29], we suggest that the severe cases (gross osteoarthritis, scarring, heterotopic bone formations, ulnar nerve problems, hardware removal, proximal radio-ulnar surgery) still should be operated with an open technique. Less severe cases with a compliant capsule and limited osteoarthritis will probably benefit from an arthroscopic capsular release. To summarize the choice between the open and the arthroscopic release should be based on the surgeon’s experience, the degree of secondary osteoarthrosis, and on the possibility for ulnar nerve release, hardware removal and proximal radial-ulnar joint surgery.

The ulnar nerve will postoperatively have an increased stress load when flexion improves. In our material only two patients had a peroperative ulnar nerve release. Knowing this, we now advocate that patients with preoperative flexion <110° should have an ulnar nerve release and be considered for peroperative antepositioning of the nerve.

Instability after arthrolysis of the elbow is rarely reported [6, 10], and we had no such cases in our material.

The main goal for the arthrolysis of the elbow is to improve ROM, not necessarily to address the pain. However, our patients report some pain release after the arthrolysis. This may be due to reduced impingement problems, and an element of denervation might as well be responsible for this beneficial side effect [10]. Our material demonstrates that satisfactory outcomes in terms of ROM, function, and pain relief endure in a long-term follow-up of open arthrolysis of the elbow. This is also in accordance with others [2, 4, 8, 10].

Rotational problems in the elbow are difficult to treat. We found that patients with bony blockings between the radius and ulna may improve the rotation after an open revision. In cases where there is no obvious focal etiology for the blocked rotation, the improvement in rotation is limited after open revision.

The strength of our paper is the significant amount of included patients and the documented outcome measures. Our liberal inclusion policy and extended use of hospitalization, postoperative brachial plexus anesthesia, and CPM machines may add some new knowledge to earlier papers addressing the stiff elbow.

## Conclusions

In compliant patients open arthrolysis is associated with reliable clinical and functional long-term outcomes in the posttraumatic stiff elbow. In patients with a gross lack of flexion, an ulnar nerve release or antepositioning should be considered peroperatively.

## Abbreviations

CPM: continuous passive motion
CRPS: complex regional pain syndrome
EQ5D: self reporting current health related quality of life state
F/E: flexion-extension sector
NSAID: nonsteroidal anti-inflammatory drug
Q-DASH: quick edition of the disability of arm, shoulder & hand score
ROM: range of motion
SD: standard deviation
VAS: visual analogue scale
